# Clinical characteristics and prognosis of cerebral venous thrombosis in Chinese women during pregnancy and puerperium

**DOI:** 10.1038/srep43866

**Published:** 2017-03-06

**Authors:** Zhu-Wei Liang, Wan-Li Gao, Li-Min Feng

**Affiliations:** 1 Department of Obstetrics and Gynaecology, Beijing Tiantan Hospital, Capital Medical University, Beijing, 100050, China

## Abstract

Due to the specific physiology associated with pregnancy and puerperium, cerebral venous sinus thrombosis (CVT) may manifest different characteristics. This study aimed to identify the clinical manifestations and prognosis of pregnancy-associated CVT. A total of 43 pregnancy-associated CVT patients were enrolled. We analysed the clinical presentations of the disease and performed a multivariate logistic regression analysis to determine which variables were associated with prognosis. Our descriptive results showed the following: 1) the incidence was 202 per 100,000 deliveries, and the mortality rate was 11.63%; 2) the most frequent symptom was headache; 3) the most frequent abnormal laboratory findings were increased levels of fibrinogen and several serum lipoproteins (including triglyceride, cholesterol, high-density lipoprotein, low-density lipoprotein, apolipoprotein A1, and apolipoprotein B); and 4) the superior sagittal sinus and transverse sinus were the most frequently affected locations. Moreover, an increased modified Rankin Scale score was positively associated with infection, seizure, intracerebral haemorrhage (ICH) and hypertensive disorders of pregnancy (HDP). Comparably, the occurrence of death was positively and significantly associated with infection, seizure and ICH. Consequently, timely diagnosis and treatment of pregnancy-associated CVT patients with infection, seizure, ICH or HDP are needed. Patients with infection, seizure or ICH have a greater risk of death.

Cerebral venous sinus thrombosis (CVT) is a relatively rare neurological emergency that occurs more often in women during pregnancy and puerperium than in the general population^1^. The incidence of CVT in the general population is as rare as 5 people per million, accounting for 0.5–1% of all strokes^2^. The reported incidence of CVT during pregnancy and puerperium in women in developed countries is 11.6 per 100,000 deliveries per year, accounting for 6–64% of pregnancy-related strokes; this rate is higher due to the different physiological profiles associated with pregnancy and puerperium^3^. In contrast, studies reporting the incidence of pregnancy-related CVT in other countries, for example, Asian countries, are rare. For example, the rate of pregnancy-related CVT in India is 450 per 100,000 deliveries^4^, which is much higher than in developed countries, and this condition accounts for 9–15% of the overall death and dependency rate of pregnant women^4^,^5^. Researchers have rarely reported the exact incidence of CVT in populations in developing countries due to the rarity and diverse manifestations of pregnancy-associated CVT. As a consequence, the crucial clinical characteristics and factors associated with the prognosis of pregnancy- and puerperium-related CVT remain unknown.

Previous research has generally suggested that the hypercoagulable state of pregnancy and puerperium is one of the main pathophysiology associated with pregnancy-related CVT^6^. The specific pathophysiological observations include iron deficiency anaemia, alterations in platelet function and in pro- and anti-thrombotic protein function and levels, and changes due to acute trauma or haemorrhage during labour and delivery^7^. As consequences of these pathophysiological changes, the clinical manifestations of pregnancy-related CVT include sudden-onset headaches, blurry vision, focal neurologic deficits, altered level of consciousness, and seizures. Notably, these symptoms are not specific and can be easily confused with the normal pregnancy state, making it difficult to diagnose and treat this disease in a timely manner, thus potentially threatening the life of pregnancy-related CVT patients and their fetuses. In contrast, if these patients are treated in a timely manner, their prognosis is better than that of non-pregnant patients with CVT^8^. Thus, to facilitate the early diagnosis of pregnancy-related CVT, there is an urgent need to characterize the clinical features of the disease.

Despite the abovementioned importance of ascertaining the clinical features of pregnancy-related CVT, the risk factors that influence the prognosis of pregnancy-related CVT patients have not been extensively studied. A meta-analysis of several recent prospective series^9^,^10^,^11^ demonstrated that the long-term predictors of poor prognosis in all CVT cases, including males and females, comprise central nervous system infection, any type of cancer, deep venous system thrombosis, intracranial haemorrhage (ICH), mental status disorders, age older than 37 years, and male gender. For pregnancy-related CVT, the possible risk factors may theoretically include caesarean section, dehydration, traumatic delivery, anaemia, increased homocysteine concentrations, and low cerebrospinal fluid pressure due to dural puncture from use of a neuraxial anaesthetic^12^,^13^. However, whether these risk factors commonly or specifically influence the outcome of CVT patients during pregnancy and puerperium remains unclear.

The aims of this study were 1) to investigate the incidence of pregnancy-related CVT in Beijing Tiantan Hospital; 2) to identify and evaluate the clinical features of pregnancy-related CVT populations, including disease onset and development, imaging features, diagnosis, treatments, and patient prognoses; and 3) to identify the magnitude of the effects of different clinical factors associated with the outcomes of CVT during pregnancy and puerperium to identify any notable elements regarding the prognosis of this disease.

## Results

### Incidence and risk factors

Between January 2006 and June 2016, a total of 43 hospitalized patients received a diagnosis of pregnancy-associated CVT. Demographic factors, comorbidities, complications, and interventions for pregnancy-associated CVT are shown in Table 1. The mean age of the patients diagnosed with CVT was 27.09 ± 4.43 years (minimum age = 20 years, maximum age = 40 years). The incidence of pregnancy-associated CVT was 202 per 100,000 deliveries. Among these patients, the incidence of puerperium-associated CVT was 80 per 100,000 deliveries, and the incidence of pregnancy-associated CVT was slightly higher at 122 cases per 100,000 births. The pregnancy-related CVT mortality rate was 11.63%. The shortest time from symptomatic onset to admission was 0.5 days after caesarean, and the longest time was 40 days postpartum. Five patients had a history of HDP, 2 had hypertension before pregnancy, 2 had a history of chronic headaches, 2 had anaemia, and 2 were recipients of progestin therapy.

### Initial symptoms and signs and abnormal laboratory findings

Within the study sample, patients showed several initial symptoms (Table 1) and abnormal laboratory findings of pregnancy-related CVT (Table 2). The binomial test revealed that the most frequent initial symptom was headache (*z*s > 3.97, *p*s < 0.0001), and the most common abnormal laboratory findings were increased serum lipoprotein and fibrinogen levels (*z*s > 2.61, *p*s < 0.01).

### Neuroimaging and other examinations

A total of 43 patients were subjected to computed tomography (CT), magnetic resonance imaging (MRI), magnetic resonance vein angiography, magnetic resonance venography (MRV) or magnetic resonance artery angiography (MRA). Patients in this sample had involvement of a number of regions (Table 1). The binomial test revealed that involvement of the superior sagittal sinus and transverse sinus was significantly more common than involvement of the straight sinus/inferior sagittal sinus, cerebral infarction, or involvement of the vein of Galen (*z*s > 2.58, *p*s < 0.01). However, the proportions of patients with these findings were not significantly different from those of patients with sinus sigmoideus or ICH.

Additionally, 6 patients exhibited papilledema, and 4 patients had an unclear boundary of the optic nerve head. The lowest and highest cerebrospinal fluid pressures were 105 cm H_2_O and 400 cm H_2_O.

### Treatment and outcome

The treatment and outcome of CVT in pregnancy and puerperium are shown in Table 3. Symptomatic treatment refers to anti-oedematous, anticonvulsive, and antihypertensive treatments. Two CVT patients in puerperium with mild symptoms did not receive anticoagulation therapy; they were given only symptomatic treatment. Patients were discharged from hospital after symptomatic improvement. A therapeutic abortion was often used to eliminate the rising endogenous oestrogen levels resulting from the pregnancy that likely triggered spontaneous coagulation. When concerns regarding the risks of anticoagulation to pregnant patients and the fetus (e.g., uterine haemorrhage, placental abruption, and miscarriage) were raised, the abortion was performed when the patients were stable and after a pause in anticoagulation therapy. Some patients have experienced dysfunction like seizures, severe visual loss due to optic atrophy, or various types of headaches and/or felt depressed or anxious after treatment. The percentage of deaths or cases was calculated based on the total number of pregnant women, except where otherwise indicated.

### Prognosis

Univariate regression analyses were conducted to identify the predictors of pregnancy-related CVT patient outcomes. The variables included in the regression were demographic variables, comorbidities, complications, and interventions. An increased modified Rankin score (mRS) was positively associated with infection, seizure, ICH, and HDP (Table 4).

Given the relatively small number of deaths (*n* = 5) in our pregnancy-related CVT sample, we also analysed the differences in the distribution of death outcomes for these four predictive variables. The chi-square test revealed that pregnancy-related CVT death was positively and significantly associated with infection, seizure, and ICH (*p*s: 0.000, 0.034, and 0.020, respectively); however, the occurrence of death was not associated with HDP (*p* = 0.191). These results indicate that among the risk factors for poor pregnancy-related CVT patient outcomes, the potential specific risk factors for patient death are infection, seizure, and ICH. Given that the patients who died had end-stage disease, they might have been in critical condition upon admission and deteriorated rapidly, resulting in death, despite receiving active therapy. Patients with this condition are at increased risk of cerebral haemorrhage, infection, and/or epilepsy, which played important roles in the deaths of the pregnancy-related CVT patients in this study (shown by the results of regression analysis).

## Discussion

In the present study, we analysed and identified the largest number of pregnancy-associated CVT cases reported to date in China. The incidence of CVT in our hospital was higher than the reported incidence in developed countries but lower than that in developing countries. Our case mortality rate of pregnancy-related CVT was 11.63%, which is consistent with previous reports. Our analysis revealed several variables associated with an increased risk of poor prognosis, including infection, seizure, ICH and HDP. Among these factors, infection, seizure and ICH were particularly associated with the death of patients with pregnancy-related CVT. These identified risk factors associated with poor prognosis may be beneficial for selecting candidates for more aggressive treatment early after diagnosis of CVT.

In the present study, the estimated incidence of CVT during the perinatal period was higher than that in previous reports on developed countries (11.6 per 100,000 deliveries) and lower than that in reports on developing countries (450 per 100,000 deliveries)^14^. This result is consistent with the conclusion of a previous study, which showed that the incidence of pregnancy-related CVT was higher in developing countries than in developed countries. Additionally, Beijing Tiantan Hospital is a referral and treatment centre for pregnant women with diagnoses of cerebrovascular disease, which may explain the increased incidence of pregnancy-related CVT in our sample.

We found that infection, seizure, ICH and HDP were associated with an increased mRS. A meta-analysis of several recent prospective series, particularly the large ISCVT cohort^9^,^10^,^11^, found that the long-term predictors of a poor prognosis for all CVT patients, including males, are CNS infection, any type of cancer, deep venous system thrombosis, ICH, a Glasgow Coma Scale score on admission of less than 9, seizure, age older than 37 years, and male gender. A study conducted in the US using the National Inpatient Sample (NIS) database examined the increased mortality rate among patients with CVT. The authors found a higher risk of death due to CVT associated with female gender (pregnant or not), white ethnicity, sepsis, hydrocephalus, ICH, and motor deficits. However, we could not reliably assess the presence of these factors in our population of patients with pregnancy-related CVT. Factors influencing the prognosis of these patients have not been extensively studied.

The present study reports a new finding: HDP are not only a risk factor for CVT but also an important factor that affects the prognosis of patients with pregnancy-related CVT. Pregnant and puerperal women are prone to intracranial venous sinus thrombosis, which may be related to the physiological state during pregnancy and puerperium^15^. Normal pregnancy is accompanied by increased concentrations of factors VII, VIII, and X and von Willebrand factor and by pronounced increases in fibrinogen. Free protein S (the active, unbound form) is decreased during pregnancy. These changes, which may not completely return to baseline until more than 8 weeks postpartum, begin with conception and result in the hypercoagulable state of pregnancy^10^,^15^. During delivery, sweating and bleeding occur, blood viscosity increases, and blood flow is slowed, which can increase the blood condensation state and cause thrombus formation. In addition, a caesarean section, older maternal age, increased vomiting, concurrent infection, reduced movement during pregnancy and puerperium, and insufficient supplementation of body fluids may increase the risk of CVT^1^,^2^,^3^,^4^,^5^. However, previous studies have not reported whether HDP affect the prognosis of patients with pregnancy-related CVT. HDP obviously increase whole blood viscosity and platelet adhesion and enhance the activation of aggregation, which together promote thrombosis. More importantly, our study found that although CVT with HDP is a prognostic risk factor, it does not influence death. This suggests that physicians should pay increased attention to CVT patients with HDP. Symptoms of headache and vomiting should alert obstetricians and neurological physicians to the presence of CVT, which may improve patient prognosis.

The present study has also clarified the effect of several other factors, including age, time from symptom initiation to admission, hypercoagulable state, infection, homocysteine level, seizures, and ICH, on the prognosis of our population. Among these factors, infection, seizure, and ICH were found to have an effect on the prognosis of patients with pregnancy-related CVT. Our findings are consistent with previous reports in a Pakistani sample that ICH and seizure are independent predictors of an unfavourable outcome at discharge^16^. Taken together, these results suggest that the effect of ICH and seizure on the prognosis of CVT patients is consistent, regardless of pregnancy status. Moreover, based on our results, septic patients with pregnancy-associated CVT are at a higher risk for a poor prognosis, regardless of the source of infection. CNS infection was a marker of poor outcomes in the ISCVT study, and sepsis had one of the strongest associations with a higher mRS and poor prognosis in our analysis. The mortality rate is high in cases of septic thrombosis of the cavernous and superior sagittal sinuses^17^. Using the NIS, Haghighi *et al*. found that the mortality rate associated with pyogenic CVT was slightly higher than that in the non-CVT group (4.55% vs. 3.52%)^18^. The outcomes were worse in a Middle Eastern series with a higher rate of CNS infection^16^. In our study, we could not determine how often sepsis was related to CNS infection. This frequency and the implications of this association deserve attention and further investigation.

The main strength of our study lies in the prognostic factors associated with CVT in patients during pregnancy and puerperium, which was not the focus in any of the previously reported cohorts. This allowed us to investigate pregnancy-related CVT more precisely and to obtain an early diagnosis, thus prompting a good prognosis.

However, this study also has several limitations. As the study was based on a hospital database, our findings are susceptible to bias or aggregation. The relatively limited number of cases recruited represents an additional limitation of the current study that inevitably introduced bias. Finally, the risk of recurrence of pregnancy-associated CVT was not evaluated in our study, and further studies are needed to clarify this issue.

In summary, we have described the clinical features of pregnancy-related CVT and examined patient prognosis. We found that infection, seizure, ICH and HDP are associated with poor prognosis. This study provides novel directions for clinicians to effectively improve the prognosis of pregnancy-associated CVT.

## Methods

### Study population

A total of 43 pregnancy-associated CVT patients were identified and enrolled in the present retrospective case study out of 21,333 deliveries between January 2003 and June 2016 at Beijing Tiantan Hospital, China. The diagnosis of CVT was based mainly on clinical manifestations and imaging results. Both obstetricians and neurologists conducted treatments and monitored disease progression. The inclusion criteria were as follows: 1) participants were pregnant or less than 6 weeks postpartum; 2) all cases of pregnancy-associated CVT were verified by neuroimaging; and 3) HDP (including pre-eclampsia, eclampsia, gestational hypertension, and chronic hypertension) were diagnosed according to the American College of Obstetricians and Gynecologists criteria^19^. Patients with CVT who were not pregnant or in puerperium were excluded from the sample. All the included patients were non-smokers, had no personal and family history of cardiovascular diseases, and had not received contraceptives (including the levonorgestrel-intrauterine system) or hormone replacement therapy. The study was approved by the Ethics Committee of Capital Medical University and conducted in accordance with the Declaration of Helsinki. Written informed consent was obtained from all participants before the commencement of the study. All methods and research activities were performed in accordance with the guidelines and regulations.

### Measures

The medical records of all participants were collected and assessed based on the following characteristics: 1) baseline demographics and clinical characteristics, including age, parity, mean hospitalization duration (days), mean duration from symptom initiation to admission (days), timing of onset in gestation, mode of delivery, risk factors, and outcomes; 2) initial symptoms, signs, and abnormal laboratory findings; 3) neuroimaging and other examination findings at evaluation; and 4) treatment and prognosis.

The hypercoagulable state of CVT was evaluated using tests for dyslipidemias (elevated low-density lipoprotein cholesterol, decreased high-density lipoprotein cholesterol, or other lipid abnormalities) and other tests (fibrinogen, homocysteine, platelets, anticardiolipin antibody, or C-reactive protein)^20^. The global outcome at discharge was assessed using the mRS: 0–2, good outcome; 3–5, poor outcome; 6, death^21^.

### Statistical analysis

All continuous variables are presented as the mean ± standard deviation. Categorical variables were also analysed. For the analysis of the primary outcome of mortality, we calculated the odds ratios and 95% confidence intervals for each demographic factor, comorbidity, complication, and intervention. A multivariate logistic regression analysis was performed to discriminate independent associations. Associations with a *p* value < 0.05 were considered statistically significant. All statistical analyses were performed using SPSS 19.0.

## Additional Information

**How to cite this article:** Liang, Z.-W. *et al*. Clinical characteristics and prognosis of cerebral venous thrombosis in Chinese women during pregnancy and puerperium. *Sci. Rep.* **7**, 43866; doi: 10.1038/srep43866 (2017).

**Publisher's note:** Springer Nature remains neutral with regard to jurisdictional claims in published maps and institutional affiliations.

## Figures and Tables

**Table 1:** Baseline demographic and clinical characteristics of pregnancy-associated CVT patients.

Variables	Values
Age (years)	27.09 ± 4.43
Parity (primipara/multipara)	35/3
Hospitalization duration (days)	18.30 ± 9.26
Time from symptom initiation to admission (days)	10.28 ± 9.83
Timing of onset in gestation	
1st, 2nd, 3rd trimester	12, 2, 3 (27.91%, 4.65%, 6.98%)
Postpartum	26 (60.47%)
Initial symptoms	
Headache	35 (81.40%)
Epileptic seizures	17 (39.54%)
Limb weakness	15 (34.88%)
Nausea/vomiting	13 (30.23%)
Disturbed consciousness	12 (27.90%)
Blurred vision	5 (11.63%)
Fever	3 (6.98%)
Neuroimaging abnormalities	
Superior sagittal sinus	27 (62.79%)
Transverse sinus	25 (58.14%)
Sinus sigmoideus	20 (46.51%)
Straight sinus/inferior sagittal sinus	12 (27.91%)
Galen vein	5 (11.62%)
Cerebral infarction	11 (25.58%)
Intracranial haemorrhage	19 (30.16)
Comorbidities	
Hypercoagulable state	5 (11.63%)
Infection	13 (30.23%)
HDP	7 (16.28%)
Homocysteine	7 (16.28%)
Complications	
Seizure	24 (55.81%)
ICH	11 (25.58%)

*Note*: Values are shown as *m* ± *SD* or *n* (%).

**Table 2:** Coagulation abnormalities in CVT patients (*N* = 43).

Characteristics	Abnormal group		Normal group		Reference interval*
	*n* (%)	*m* ± *SD*	*n* (%)	*m* ± *SD*	
SLP					
TG (mmol/L)	8 (18.60%)	4.78 ± 1.54	35 (81.40%)	1.17 ± 0.42	0.50–1.70
CHO (mmol/L)	28 (65.12%)	6.72 ± 3.43	15 (34.88%)	4.24 ± 0.45	3.20–5.17
HDL (mmol/L)	3 (6.98%)	2.86 ± 0.65	40 (93.02%)	1.40 ± 0.24	1.00–1.80
LDL (mmol/L)	24 (55.81%)	4.64 ± 1.06	19 (44.19%)	2.14 ± 0.32	1.50–3.10
APO-A1 (mmol/L)	15 (34.88%)	3.58 ± 1.32	28 (65.12%)	1.40 ± 0.16	1.20–1.80
APO-B (mmol/L)	22 (51.16%)	5.46 ± 2.09	21 (48.84%)	0.86 ± 0.12	0.60–1.14
FBG (g/L)	25 (58.14%)	4.64 ± 0.32	18 (41.86%)	2.96 ± 0.84	2.00–4.00
HCY (μmol/L)	9 (20.93%)	43.56 ± 7.85	34 (79.07%)	12.57 ± 2.15	≤ 15
PLT (*10^9^/L)	13 (30.23%)	365.65 ± 30.64	30 (69.77%)	169.78 ± 15.79	100–300
ACL	2 (4.65%)	—	41 (95.35%)	—	negative
CRP (mg/L)	5 (11.63%)	48.95 ± 14.56	38 (88.37%)	1.23 ± 0.45	0.00–2.87

*Note*: Abbreviations: SLP, serum lipoprotein; TG, triglyceride; CHO, cholesterol; HDL, high-density lipoprotein; LDL, low-density lipoprotein; APO-A1, apolipoprotein A1; APO-B, apolipoprotein B; FBG, fibrinogen; HCY, homocysteine; PLT, platelets; ACL, anticardiolipin antibody; CRP, C-reactive protein. *The normal range of coagulation abnormalities at Beijing Tiantan Hospital.

**Table 3:** Treatments and outcomes of CVT patients (*N* = 43).

Characteristics	CVT patients	
	Pregnancy (*n* = 17)	Puerperium (*n* = 26)
Treatments		
Anticoagulation	17 (100%)	24 (92.31%)
Thrombolysis and thrombectomy with anticoagulation	2 (11.76%)	1 (3.85%)
Symptomatic treatment without anticoagulation	0 (0.00%)	2 (7.69%)
Obstetric outcomes		
Caesarean delivery	4 (23.53%)	12 (46.15%)
Vaginal delivery	0 (0.00%)	13 (50.00%)
Induced labour	1 (5.88%)	0 (0.00%)
Abortion	8 (40.06%)	1 (3.85%)
Death before obstetric treatment	4 (23.53%)	0 (0.00%)
Outcomes		
Death	4 (23.53%)	1 (3.85%)
Recovered	9 (52.94%)	18 (69.23%)
Dysfunction	4 (23.53%)	7 (26.92%)

*Note*: ^1^dead. ^2^6 weeks and an IVF postoperative twin pregnancy.^3^, Among the patients who died, 4 died in early pregnancy (1 case at 8 weeks and 3 cases at 10 weeks), and 1 died in puerperium (2 weeks). The gravidity was 2 for 3 patients and 1 for 2 patients. This pregnancy was the second pregnancy in 3 patients, the first pregnancy in 2 patients.

**Table 4:** Predictors influencing the prognosis of pregnancy-related CVT patients.

Predictors	OR (95% CI)	*p* values
Demographics		
Age (years)	0.02 (−0.06, 0.10)	0.67
Time from symptom initiation to admission (days)	0.00 (−0.04, 0.03)	0.88
Risk factors		
Hypercoagulable state	0.25 (−0.43, 0.93)	0.46
Infection	1.85 (1.11–2.60)	0.00
Homocysteine	0.41 (−0.51, 1.32)	0.38
Seizure	1.00 (0.32–1.68)	0.01
ICH	1.26 (0.56–1.96)	0.00
HDP	1.26 (0.36–2.15)	0.01
